# Peripheral lncRNA NEAT-1, miR374b-5p, and IL6 panel to guide in COVID-19 patients’ diagnosis and prognosis

**DOI:** 10.1371/journal.pone.0313042

**Published:** 2024-12-27

**Authors:** Marwa A. Ali, Olfat G. Shaker, Eman M. Ezzat, El Shaimaa Gomaa Ali, Mahmoud I. Aboelor, Mona I. Ahmed, Essam A. Hassan, Doaa Y. Ali, Heba Mostafa Ahmed, Abeer A. Khalefa, Rasha A. Hussein, Ahmed Fathy Elkhateeb, Esam Ali Mohamed

**Affiliations:** 1 Faculty of Medicine, Department of Medical Biochemistry and Molecular Biology, Fayoum University, Fayoum, Egypt; 2 Department of Biomedical Sciences, College of Medicine, King Faisal University, Alhasa, Saudi Arabia; 3 Faculty of Medicine, Department of Medical Biochemistry and Molecular Biology, Cairo University, Cairo, Egypt; 4 Faculty of Medicine, Department of Internal Medicine, Fayoum University, Fayoum, Egypt; 5 Faculty of Medicine, Department of Medical Microbiology and Immunology, Fayoum University, Fayoum, Egypt; 6 Faculty of Medicine, Department of Radio-Diagnosis, Fayoum University, Fayoum, Egypt; 7 Faculty of Medicine, Department of Chest Disease, Fayoum University, Fayoum, Egypt; 8 Faculty of Medicine, Department of Tropical Medicine, Fayoum University, Fayoum, Egypt; 9 Faculty of Medicine, Department of Clinical and Chemical Pathology, Fayoum University, Fayoum, Egypt; 10 Faculty of Medicine, Department of Physiology, Zagazig University, Zagazig, Egypt; 11 Faculty of Medicine, Department of Medical Microbiology and Immunology, Zagazig University, Zagazig, Egypt; 12 Faculty of Medicine, Department of Critical Care, Fayoum University, Fayoum, Egypt; Pennsylvania State University Hershey Medical Center, UNITED STATES OF AMERICA

## Abstract

**Background:**

The SARS-CoV-2 virus’s frequent mutations have made disease control with vaccines and antiviral drugs difficult; as a result, there is a need for more effective coronavirus drugs. Therefore, detecting the expression of various diagnostic biomarkers, including ncRNA in SARS-CoV2, implies new therapeutic strategies for the disease.

**Aim:**

Our study aimed to measure NEAT-1, miR-374b-5p, and IL6 in the serum of COVID-19 patients, demonstrating the correlation between target genes to explore the possible relationship between them. Also, the association between target genes and patients’ clinical findings and radiological severity indices will be explored.

**Patients and methods:**

The current study included 48 COVID-19-infected individuals and 40 controls. Quantitative real-time PCR (qPCR) was performed to detect lncRNA *NEAT-1* and *miRNA374b-5p* fold change (FC) in the participants’ sera. Enzyme-Linked Immune Sorbent Assay (ELISA) is used to detect *IL6*.

**Results:**

Our results showed statistical significance with lower levels of (*NEAT-1*) [ median (range) = 0.08 (0.001-0.602)], and (*miR374b-5p*) [ median (range) = 0.14 (.01-7.16)] while higher *IL-6* levels [ median (range) = 41.3 (7.2-654) pg/ml] when compared to controls with p-value <0.001. Serum level of *NEAT-1* correlates negatively with *IL-6* level (*r* = -.317, P = .008). ROC curve analysis revealed that sensitivity and specificity tests for *NEAT-1* and IL-6 levels in the diagnosis of cases illustrated a sensitivity of (100% and 97.9%) and a specificity of (85% and 100%) at cut-off values (0.985 and 12.55), respectively. In comparison, *miR374b-5p* showed sensitivity and specificity of around 85% in distinguishing COVID-19 patients from controls. No significant association was detected between target genes and radiological severity indices.

**Conclusions:**

Our study is the first to detect decreased *NEAT-1* and *miR374b-5p* expression in COVID-19 patients’ serum. There was also an increase in *IL6* levels. There is a negative correlation between *NEAT-1* and *IL6* in COVID-19 patients.

## Introduction

The COVID-19 pandemic, caused by the SARS-CoV-2 virus (severe acute respiratory syndrome coronavirus 2), presents an ongoing challenge to the medical and research community. Over 775 million confirmed cases and over seven million deaths have been reported globally by June 2024 [1].

Several studies reported the role of ncRNAs in coronavirus infection. Among these ncRNAs are lncRNAs that are nearly 200 nucleotides long with no translational activity; instead, these lncRNAs can interact with various molecules, such as DNA, proteins, and other RNAs. Additionally, they may modify targeted mRNA expression by sponging microRNAs (types of ncRNA molecules with 21-25 nt in length) for the same binding sites, as proposed by competing endogenous RNA (ceRNA hypothesis) [2].

One of the lncRNAs, nuclear paraspeckle assembly transcript 1 (*NEAT-1*), has been shown to play a role in the inflammatory activities of immune cells, including macrophages and monocytes [3]. Previous literature showed that *NEAT-1* is overexpressed in the peripheral blood of CORVID-19 patients [4], while others showed that its expression is exclusive to tissues [5]. Furthermore, some articles showed that *NEAT-1* is differentially expressed between different stages of disease severity [5,6]. However, others showed that there are no significant differences in *NEAT-1* expression between moderate and severe stages of the disease [4,7] or between the acute and post-acute phases of COVID-19 infection [8]. Meanwhile, ***Meydan et al*., *2020*** article showed that *NEAT-1* is down-regulated in lung cells infected with SARS-CoV2 compared to normal lung cells or lung cells infected with other respiratory viral infections such as the influenza virus (H1N1) [6]. These controversial results reflect the various *NEAT-1* functions through targeting different noncoding RNA and different protein-coding RNA genes in different stages and severity of COVID-19 infection [3]

Amongst this noncoding RNA that is targeted by *NEAT-1* is *miR374b-5p*, which is in 2023 recent research demonstrating that *NEAT-1* may serve as the ceRNA of *miR-374b-5p* in the development of osteoarthritis [9] ***Huang et al*. *2023*** reported that silencing of *NEAT-1* increases *miR374b-5p* expression [9]. Furthermore, in another 2023 bioinformatic analysis paper, *miR374b* can inhibit the RNA-dependent RNA polymerase enzyme (RdRp), which is crucial for the life cycle of the SARS-CoV2 virus that leads to decreased inflammation in COVID-19 infection and maybe a new therapeutic approach to coronavirus control [10].

Among the protein-coding RNAs targeted by *NEAT-1* is *IL6* [10]. *IL6* is an essential cytokine that increases in patients with COVID-19 at all stages of the disease and at all severity levels [11] that ends with cytokine storm syndrome [12–14], and fatal acute respiratory distress syndrome and other organ failure [13]. *IL-6* is also a key cytokine target for therapy in COVID-19 [14,15].

Recently, it was found that *IL6* is a target for *miR-374* family members; ***Sanchez et al*., *2022*** demonstrated that by in vivo and invitro evidence, *miR-374a-5p* expression was inversely correlated with some of its targets, including *IL6* and other inflammatory genes in inflammatory bowel disease [16]. Additionally, ***Zhao et al*., *2022*** reported that the loss of *miR-374c-5p* negatively regulates *IL6* in unexplained recurrent spontaneous abortion [17]. Furthermore, ***Huang et al*., *2023*** found that the loss of *NEAT-1* or the upregulation of *miR-374b-5p* dramatically accelerated apoptosis, led to the arrest of G1 / S, and promoted the secretion of inflammatory cytokines (*TNF-α*, *IL-1β*, and *IL-6*) in lipopolysaccharide (LPS) induced chondrocytes [9].

Our study aimed to measure NEAT-1, miR-374b-5p, and IL6 in the serum of COVID-19 patients, demonstrating a correlation between target genes to explore the possible relationship between them. Also, to explore the association between target genes and clinical findings of the patients, as well as radiological severity indices.

## Materials and methods

### Ethical approval and sample collection

EL-Fayoum Ethical Committee no (R 239, Session No 96) approved the current case-control study. After all participants were informed of the aim and procedure of the study, they assigned written consent. This study followed the ethical rules of Helsinki. COVID-19 patients were enrolled from the Chest, Internal Medicine, Tropical Medicine, and Critical Care departments of Fayoum University Hospital, El Fayoum, Egypt. All blood samples were collected from Jul. 15, 2023, to 25 ^th^ Sep 2023. All practical experiments were done in the Medical Biochemistry, Microbiology, and Clinical Pathology departments at Fayoum University, Al Fayoum, Egypt.

### Participants

The current study included 48 COVID-19-infected individuals and 40 healthy subjects. Nasopharyngeal swabs were collected from all patients and used for reverse transcriptase PCR (RT-PCR) for detection of the virus; the initial RT-PCR test revealed that 38 patients were positive while 10 were negative RT-PCR but, by repeating RT-PCR, all patients were positive. Exclusion criteria include co-infected with another viral infection like HBV, HIV, HCV, or CMV or with TB or other superimposed bacterial chest infections detected by sputum discoloration and confirmed by culture were excluded from the study. Also, pregnant females were excluded.

History taking, routine physical examination, arterial blood gases, and laboratory tests were performed for all patients. CT and chest radiographs were done for all patients. Diagnosis of COVID-19 by considering a combination of factors, including a physical examination and review of symptoms by a healthcare professional, imaging tests such as X-rays or CT scans, CO-RADs (COVID-19 Reporting and Data System) or CT-RSNA, and /or positive reverse transcriptase real-time (RT-PCR) [18,19]. Forty healthy controls were involved in this study without any acute infection or chronic disease. Negative RT-PCR of nasopharyngeal swabs confirmed the eligibility of healthy individuals.

### Radiological scoring system of COVID-19 Severity

CT-TSS (CT total severity score), a quantitative clinical evaluation, will grade each lobe of the five lung lobes from 1 to 4 according to the affected portion in the following way: 0 = (0%), 1 = (1-25%), 2 = (26-50%), 3 = (51-75%), or 4 = (76-100%). The total severity scores (TSS) were calculated by summing up the grade from each lobe, and the TSS score ranged from 0 to 20. The patients were divided into four groups: none (0), mild (1–5), moderate (6–10), and severe (11–20).

Mild and Moderate patients were admitted to inpatient rooms, while severe cases were admitted to the Critical Care department of Fayoum University Hospital and were put on mechanical ventilation.

The Chest X-Ray Scoring System (CXR score 18) used in this study involved dividing the lungs into six regions using two lines. Each region was assigned a score of 0 to 3 based on the extent of lung lesions observed. A score of 0 indicated normal lung appearance, 1 indicated interstitial infiltrates, 2 indicated a combination of interstitial and alveolar infiltrates (with interstitial dominance), and 3 indicated a combination of alveolar and interstitial infiltrates (with alveolar dominance). The scores for the six lung zones were added, resulting in a total score of 0 to 18. The patients were then classified into four groups based on their overall CXR score: normal (score of 0), mild (score of 1-6), moderate (score of 7-12), and severe (score of 13-18). This new scoring system allows for the determination of disease severity in COVID-19 patients [20].

### Preparation of serum samples

A total of five milliliters of blood were collected from each participant. Out of the five milliliters, two milliliters were collected in EDTA tubes. The remaining three milliliters were centrifuged to separate the serum and then used to assess the relative expression of *miR-374b-5p* and *NEAT-1* through RT-PCR. Additionally, the serum protein level of *IL6* was measured using ELISA. Various other tests, including CBC, serum creatinine, urea, alanine aminotransferase (ALT) and aspartate aminotransferase (AST), D-dimer, lactate dehydrogenase (LDH), albumin, serum Na and K and C-reactive protein (CRP) levels, were also examined.

### Total RNA isolation and complementary DNA (cDNA) synthesis

The Qiagen kit from Valencia, CA, USA, was used to extract RNA from the serum. In summary, 200 μL of the serum sample was mixed with 1 mL of QIAzol lysis reagent and incubated at room temperature for 5 minutes. The mixture was then subjected to chloroform phase separation. The upper aqueous phase was combined with 100% ethanol and transferred to RNeasy Mini spin columns placed in collection tubes. The columns were centrifuged at room temperature at a speed of 8000 xg for 15 seconds. Subsequently, the RNA was eluted from the columns. The eluted RNA was then quantified, and its purity was assessed using the NanoDrop® (ND)-1000 spectrophotometer from NanoDrop Technologies, Inc. in Wilmington, USA. The extracted RNA was subjected to reverse transcription using the miScript II RT kit from Qiagen, located in Valencia, CA, USA. The reverse transcription reaction was performed in a final volume of 20 μL.

### Quantitative real-time PCR (qPCR) for the detection of lncRNA and miRNA

Serum expression levels of the studied lncRNA *NEAT-1* and *miR374b-5p* were evaluated using *GAPDH* and *SNORD 68* as internal controls, respectively. Following the manufacturer’s instructions, we used primers for NEAT-1, miR374b-5p, GAPDH, SNORD 68 (Table 1), and the Maxima SYBR Green PCR kit (Thermo, USA). For a qPCR reaction, a 20-μl mixture was prepared (10 μl master mix + 1 μl forward primer + 1 μl reverse primer + 2.5 μl cDNA + 5.5 μl RNAase-free water). The experiment was executed by the Rotor-Gene Q System (Qiagen) with the following cycling conditions: 95°C for 10 min, followed by 45 cycles at 95°C for 15 s and 60°C for 60 s.

**Table 1:** Primers used in q-PCR.

*Gene*	Forward primer	Reverse Primer
*NEAT-1*	5′-TGGCTAGCTCAGGGCTTCAG-3′	5′-TCTCCTTGCCAAGCTTCCTTC–3′
*GAPDH*	5′-CTGACTTCAACAGCGACACC-3′	5′-TAGCCAAATTCGTTGTCATACC-3′
*MiR374b-5p*	5′–GCGCGATATAATACAACCTGC-3′	5′–AGTGCAGGGTCCGAGGTATT–3′
*SNORD68*	5′–CGCGTGATGACATTCTCC-3′	5′–GATGGAAAAGGGTTCAAATGT–3′

### Gene expression analysis using real-time PCR

Fold changes (FC) of target genes (*NEAT-1* and *miR374b-5p*) were measured using the 2^-ΔΔCt^ equation. First, we subtract the Ct values of *SNORD 68* from the Ct values of the *miR374b-5p* and the Ct values of *GAPDH* from the Ct of *NEAT-1*. Second, the ΔΔCt was determined by subtracting the ΔCt of controls from the ΔCt of patients [21].

### Human interleukin 6 (IL-6) ELISA Kit

We used an Enzyme-Linked Immune Sorbent Assay (ELISA) kit from Bioassay Technology to detect human interleukin 6 based on Biotin double antibody sandwich technology. Incubate wells already pre-coated with the interleukin-6 (*IL-6*) monoclonal antibody. Next, biotin-labeled anti-IL-6 antibodies with streptavidin-HRP are combined to form an immune complex. After incubation and washing, remove any enzymes that remain unbound. Combine substrates A and B. The solution will then turn blue and yellow because of the acidic effect. The solution shades and the concentration of human interleukin 6 (*IL-6*) correlate positively.

### Statistical analysis

The collected data were organized and coded to facilitate data manipulation. It was then double-entered into Microsoft Access, a database management system. Data analysis was performed using the Statistical Package for the Social Sciences (SPSS) software version 22, running on Windows 7 (SPSS Inc., Chicago, IL, USA). A simple descriptive analysis was conducted for qualitative data, presenting the data as numbers and percentages. For quantitative parametric data, central tendency was measured using arithmetic means, which provide an average value. The dispersion of the data was assessed using standard deviations. A comparison between quantitative parametric data was performed by using an independent samples t-test between two groups and a one-way ANOVA test if there were more than two groups. For quantitative non-parametric data, the Kruskal-Wallis test was performed to compare more than two independent groups, and the Mann-Whitney test was used for more than two independent groups. For qualitative data, the Chi-square test is used. Bivariate Spearman Correlation test to explore the possible correlation between quantitative non-parametric variables. ROC curve "Receiver Operating Characteristic" was executed. The P-value< 0.05 is a significant value.

### Sample size

The sample size was calculated using the G-Power version 3.1.7 (Institute of Experimental Psychology, Heinrich Heine University, Dusseldorf, Germany). The minimum sample size of the patients was 47. Effect size: 0.60, based on previous research findings. The two-sided (two tails) type I error is 0.05, with 80% power.

## Results

Raw data supporting the results were shown in (S2 Text).

### Demographic basic data of studied subjects

The detailed description of basic medical history, full examination laboratory findings and treatment regimens are shown in **Tables A-E in S1 Text. Table 2** showed insignificant differences between cases and controls regarding age and sex.

**Table 2:** Demographic characteristics of COVID-19 and healthy controls.

Variables	Cases (N = 48)		Control (N = 40)		P-value
**Age (years)**					
Mean ±SD	61.2	10.8	56.1	9.7	0.07
**Sex**					
Male	28	58.3%	12	30%	0.06
Female	20	41.7%	28	70%	

### Expression profile of fold change (*NEAT-1*), fold change *miR374b-5p*, and *IL-6* in cases and controls

**Fig 1:**
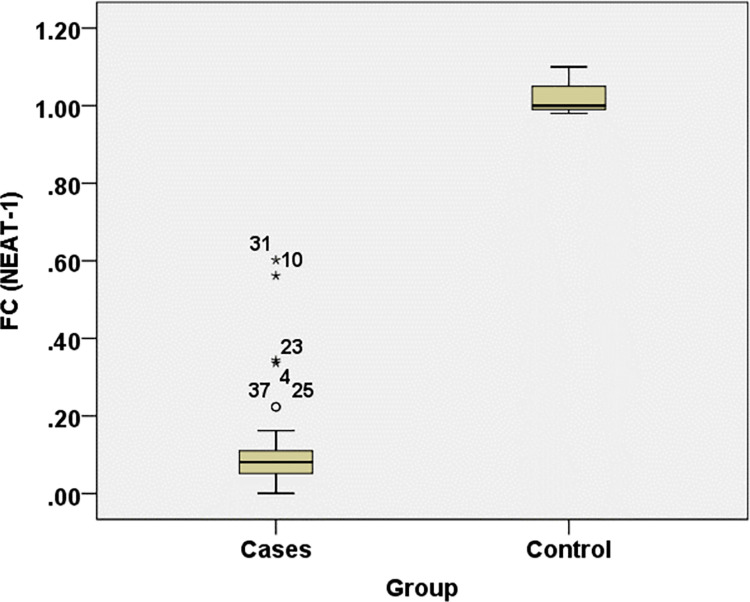
FC (NEAT1) level comparisons in different study groups.

**Fig 2:**
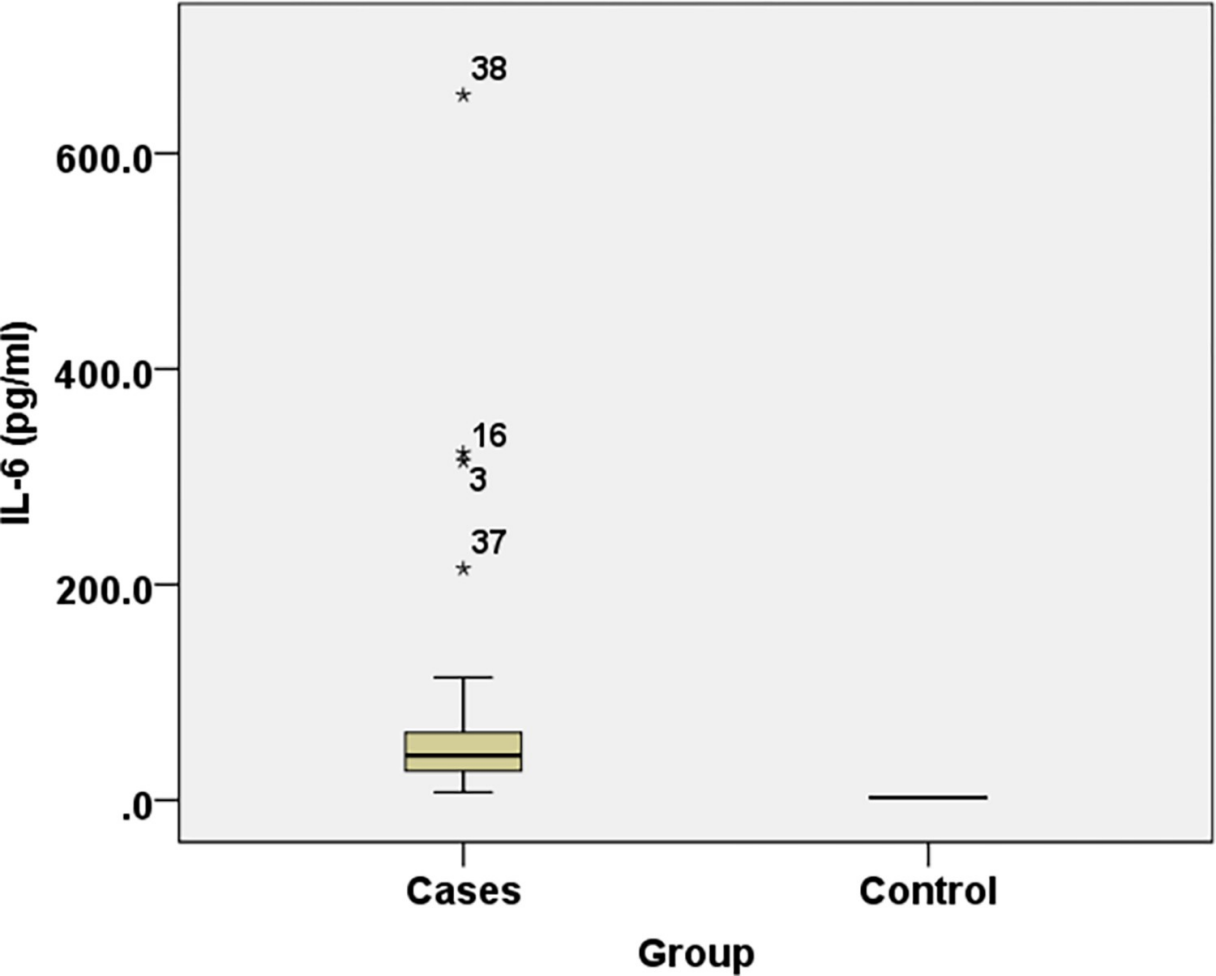
Comparisons of *IL-6* levels in different study groups.

**Fig 3:**
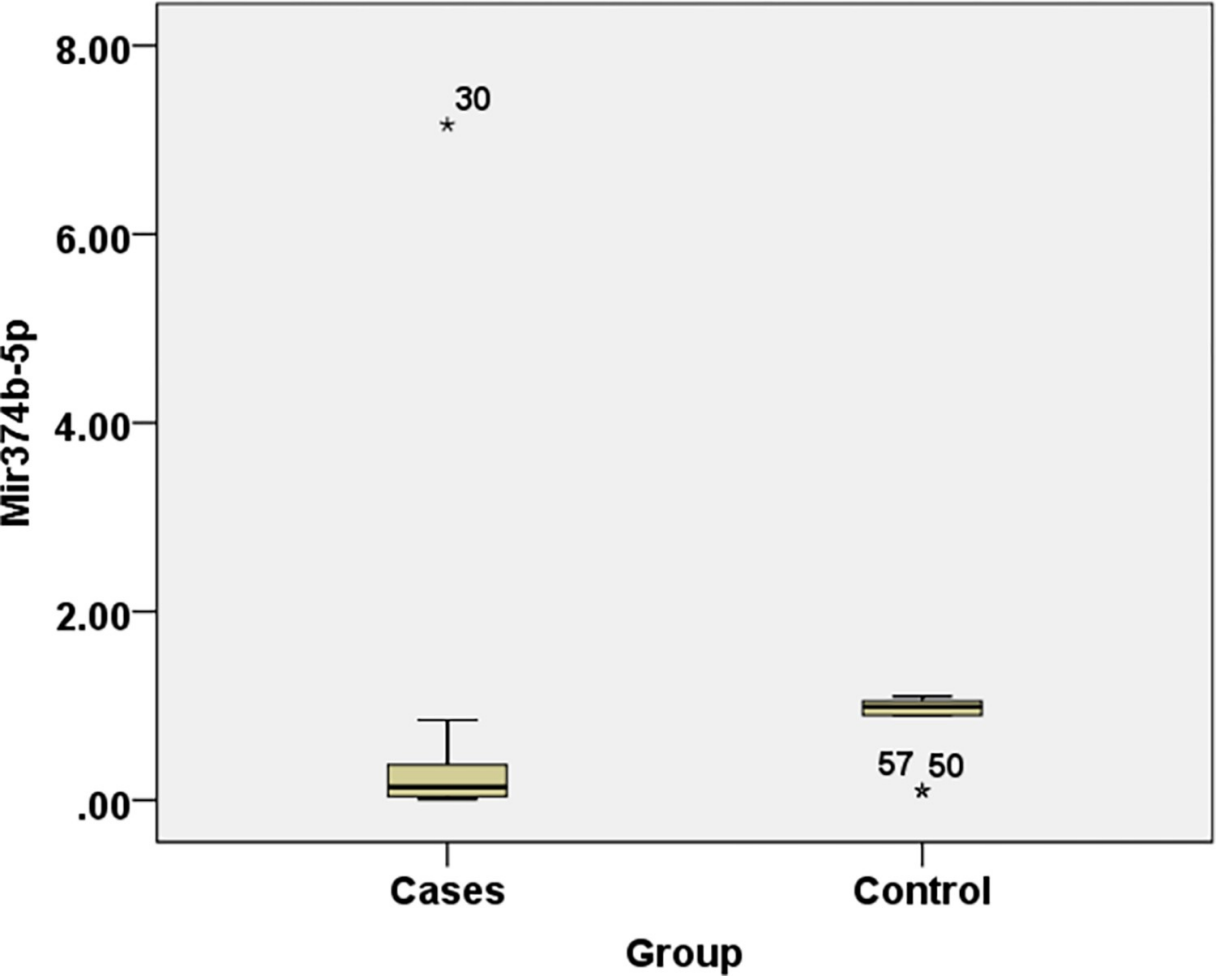
Comparisons of FC (*miR374b-5p*) levels in different study groups.

Cases showed statistical significance with lower levels of FC (*NEAT-1*) and FC (*miR374b-5p*) while higher *IL-6* levels when compared to controls with P-value <0.001 **(Table 3) (Figs 1–3).**

**Table 3:** Fold change of *NEAT1*, *miR374b-5p*, and *IL-6* levels in COVID-19 and healthy controls.

Studied parameter	COVID-19 (N = 48)		Healthy subjects (N = 40)		P-value
	Median	Range	Median	Range	
**FC (*NEAT1*)**	0.08	0.001-0.602	0.99	0.98-1.1	<0.001*
***IL-6* (pg/ml)**	41.3	7.2-654	2.4	1.1-3.4	<0.001*
**FC (*mir374b-5p)***	0.14	0.01-7.16	0.99	0.98-1.1	<0.001*

FC; fold change.

### Correlation between *NEAT-1*, *miR374b-5p* and *IL6* levels

**Fig 4:**
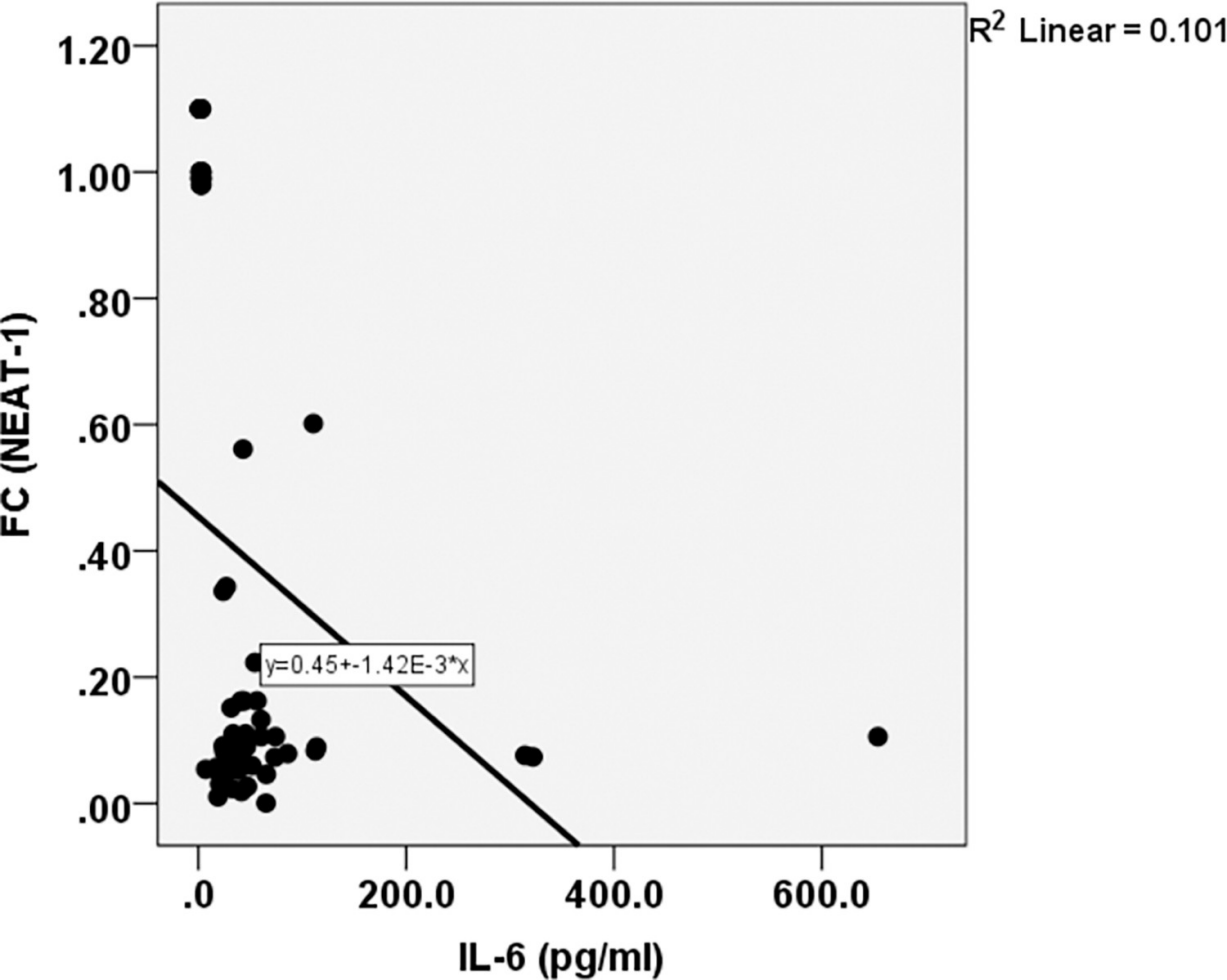
Spearman correlation between *NEAT1* and *IL6* in cases.

Fold change of *NEAT-1* correlates negatively with IL-6 level (*r* = -.317, P = .008) **(Table 4) (Fig 4).**

**Table 4:** Correlation analyses between studied genes.

Variable		FC (*NEAT-1*)	*IL-6* (pg/ml)	FC *(miR374b-5p)*
**FC (*NEAT-1*)**	r	1	-.317**	.211
	P value		**.008**	.084
***IL-6* (pg/ml)**	r	-.317**	1	-.125
	P value	**.008**		.312
**FC *(miR374b-5p)***	r	.211	-.125	1
	P value	.084	.312	

**. Correlation is significant at the 0.01 level (2-tailed).

### Comparisons of FC (*NEAT-1*), *IL-6*, and *miR374b-5p* levels in different demographic and medical, CT findings, and clinical characteristics between COVID-19 patients

No significant differences were detected between *NEAT-1*, *IL-6* or *miR374b-5p* levels and sex, comorbidities, and treatment types. **(Table 5A).** There was a statistically significant lower median of *NEAT-1* level among cases with GGO (median (range) was 0.06 (0.001-0.11) versus 0.09 (0.01-0.60) in cases with no GGO), and higher *IL-6* level among cases with grade 4 CORADS degree (62.9 versus nearly 40 for grade 3 and 5) with P-value <0.05, but no difference in other CT findings as regards levels of *NEAT-1* and *IL-6* **(Table 5B).**

**Table 5:** A. Comparisons of FC (*NEAT1*), *IL-6*, and FC (*miR374b-5p)* levels in different demographic and medical characteristics among cases. **B.** Comparisons of FC (*NEAT--1*), *IL-6*, and *miR374b-5p* levels in different CT findings among cases.

Variables	FC (*NEAT-1*)		P-value	*IL-6* (pg/ml)		P-value	FC *(miR374b-5P)*		P-value
	Median	Range		Median	Range		Median	Range	
**Sex**									
Male	0.083	0.001-0.62	0.20	39.9	17.9-322	0.11	0.12	0.01-0.85	0.12
Female	0.077	0.019-0.336		43.5	7.2-654		0.31	0.02-7.16	
**Comorbidities**									
DM	0.098	0.01-0.60	0.06	43.1	7.2-654	0.06	0.14	0.01-7.16	0.77
HTN	0.93	0.01-0.34	0.46	41.1	7.2-654	0.87	0.34	0.01-7.16	0.16
Coronary disease	0.11	0.04-0.22	0.54	33.5	22.1-214.7	0.90	0.08	0.01-0.35	0.41
CKD	0.08	0.04-0.60	0.71	110.7	22.8-112.7	0.48	0.12	0.01-0.57	0.66
Other comorbidities	0.09	0.01-0.60	0.21	39.9	7.2-654	0.26	0.12	0.01-0.82	0.42
**Treatment types**									
Ivermectin	0.07	0.04-0.34	0.94	27.4	23.6-322	0.76	0.05	0.03-0.14	0.16
Remedesevir	0.08	0.001-0.34	0.87	32.9	22.1-322	0.41	0.11	0.01-0.85	0.79

Variables	*FC (NEAT-1)*		P-value	IL-6 (pg/ml)		P-value	FC (miR374b-5p)		P-value
	Median	Range		Median	Range		Median	Range	
CT findings									
Bilateral GGO	0.08	0.001-0.60	0.99	41	47.2-654	0.54	0.17	0.01-7.16	0.082
Rt lung affection	0.09	0.05-0.11		43.1	38.7-74.2		0.04	0.02-0.05	
GGO	0.06	0.001-0.11	0.01*	47.2	7.2-322	0.77	3.23	0.01-7.16	0.85
Consolidation	0.12	0.04-0.34	0.30	29.2	22.8-60.3	0.28	0.035	0.02-0.12	0.63
Mixed GGO and consolidation	0.09	0.01-0.60	0.10	41.3	17.9-654	0.75	0.19	0.01-0.82	0.26
Plural effusion	0.08	0.01-0.16	0.52	46.7	22.1-654	0.10	0.85	0.1-7.16	0.15
Nodules	0.08	0.01-0.33	0.45	42.3	20.6-654	0.71	0.12	0.01-0.82	0.29
Pattern bronchial dilatation (ETC)	0.07	0.01-0.11	0.48	314.2	41.5-654	0.06	0.11	0.02-0.35	0.63
**Distribution**									
Central distribution	0.08	0.03-0.15	0.61	47.2	22.8-74.2	068	0.05	0.02-7.16	0.37
Peripheral distribution	0.08	0.02-0.22	0.88	32.1	20.6-112.7	0.25	0.17	0.02-0.82	0.49
Mixed distribution	0.07	0.001-0.56	0.82	41.5	17.9-654	0.64	0.21	0.01-0.85	0.61
**Co-RADS degree**									
Grade 3	0.09	0.05-0.15	0.98	39.9	31.5-74.2	0.04*	0.35	0.02-0.08	1.000
Grade 4	0.08	0.02-0.22		62.9	7.2-654		0.34	0.02-0.72	
Grade 5	0.07	0.001-0.60		33.2	17.9-322		0.17	0.01-7.16	
**CT-RSNA**									
Typical	0.07	0.001-0.60	0.96	37.4	7.2-322	0.42	0.19	0.01-7.16	0.21
Undetermined	0.08	0.02-0.15		41.5	31.5-654		0.05	0.02-0.57	

GGO, Ground glass opacity; CO-RADS, COVID-19 Reporting and Data System; CT-RSNA, Radiological Society of North America.

No statistically significant difference was reported between patients regarding FC (*NEAT-1*), FC (*miR374b-5p*), and *IL-6* levels in different clinical data **(Table 6).**

**Table 6:** Comparisons of FC (*NEAT-1*) and *IL-6*, FC (*miR374b-5p*) levels in different clinical characteristics among cases.

Variables	FC (*NEAT-1*)		P-value	*IL-6* (pg/ml)		P-value	FC *(miR374b-5p)*		P-value
	Median	Range		Median	Range		Median	Range	
**Inotropes**									
No	0.08	0.001-0.602	0.45	43.1	7.2-645	0.09	0.17	0.01-7.16	0.34
Yes	0.074	0.023-0.343		26.9	23.6-322		0.06	0.03-0.69	
**ICU admission**									
No	0.082	0.019-0.561	0.80	41.5	7.2-322	0.78	0.21	0.02-0.82	0.63
Yes	0.078	0.001-0.602		41.1	17.9-654		0.14	0.01-7.16	
**Type of ventilation**									
Deep NIV	0.073	0.023-0.343	0.79	26.9	23.6-322	0.41	0.06	0.03-0.69	0.69
Mask/nasal cannula	0.089	0.019-0.602		43.1	7.2-314.2		0.14	0.01-0.82	
MV	0.078	0.001-0.336		41.1	18.8-654		0.17	0.01-7.16	
CPAP	0.059	0.046-0.162		43.9	33.1-65.5		0.24	0.02-0.35	
**PCR**									
Negative	0.086	0.031-0.343	0.77	35.9	20.6-322	0.75	0.05	0.02-0.82	0.35
Positive	0.078	0.001-0.602		42.3	7.2-654		0.18	0.01-7.16	

NIV, non-invasive ventilation; MV, mechanical ventilation; CPAP, continuous positive airway pressure.

### Correlation between FC (*NEAT-1*), *IL-6*, FC (*miR374b-5p)* levels & clinical and laboratory data among cases

There was no statistically significant correlation among cases between FC (*NEAT-1*), FC (*miR374b-5p*), and *IL6* levels & vital data **(Table 7).**

**Table 7:** Correlation between FC (*NEAT-1*), *IL-6* levels, FC (*miR374b-5p*), and clinical data among cases.

Variables	FC (*NEAT-1*)		*IL-6* (pg/ml)		*FC (miR374b-5p)*	
	r	P-value	r	P-value	r	P-value
SBP	0.22	0.14	-0.04	0.77	0.04	0.78
DBP	0.17	0.24	0.04	0.78	0.08	0.60
Temperature	-0.05	0.73	0.001	0.99	-0.003	0.98
RR	-0.02	0.89	-0.09	0.56	0.27	0.07
HR	-0.15	0.31	-0.05	0.71	-.017	0.91
O2 on RA	-0.02	0.90	0.09	0.59	-.306	0.08
O2 on oxygen	0.01	0.93	0.02	0.90	-.171	0.25
GCS	-0.06	0.66	-0.19	0.18	.015	0.92

SBP, systolic blood pressure; DBP, diastolic blood pressure; RR, respiratory rate; HR, heart rate; GCS, Glasgow Coma Scale; O2 on RA, oxygen on room air; FC, Fold Change.

There was a statistically significant positive correlation between FC level (*NEAT-1*) and level of RBG and a statistically significant negative correlation with WBCS, ALT, AST and creatinine. The *IL-6* level showed a statistically significant positive correlation with ALT, AST, and creatinine (p-value <0.05). *MiR374b-5p* showed a significant positive correlation with serum Na while a significant negative correlation with HB, MCV, MCH, and MCHC **(Table 8).**

**Table 8:** Correlation between FC (*NEAT-1*), *IL-6*, *miR374b-5p* levels, and laboratory data among cases.

Variables	FC (*NEAT-1*)		*IL-6* (pg/ml)		FC *(miR374b-5p)*	
	*r*	P-value	*r*	P-value	*r*	P-value
Age	-0.2	0.09	0.17	0.16	.085	0.58
Duration of hospital stay (days)	0.04	0.75	0.022	0.11	.145	0.33
Serum Na	0.04	0.81	0.09	0.57	.297*	0.042*
Serum K	-0.05	0.72	0.24	0.09	-.105	0.48
RBG	0.29*	0.04*	0.17	0.23	.001	0.99
RBCS	0.13	0.37	0.01	0.93	-.053	0.72
HB (g/dl)	0.18	0.13	-0.11	0.34	-.318*	0.028*
Hematocrit (%)	0.11	0.7	0.04	0.76	-.267	0.06
MCV (fl)	0.08	0.58	0.12	0.41	-.396*	0.005*
MCH (pg)	0.03	0.83	0.09	0.53	-.414*	0.003*
MCHC (g/dl)	-0.05	0.76	-0.06	0.65	-.371*	0.012*
RDW (%)	-0.13	0.44	-0.18	0.26	.199	0.22
Platelet Count	-0.005	0.96	0.002	0.98	-.035	0.81
Mean platelet. Volume	-0.20	0.26	-0.25	0.16	-.047	0.79
Total WBCs	-0.29*	0.01*	0.22	0.07	-.251	0.08
Neutrophil	-0.06	0.72	-0.09	0.58	-.192	0.26
Lymphocyte	-0.006	0.96	0.23	0.10	-.159	0.28
Monocyte	-0.13	0.68	0.05	0.87	-.442	0.15
Basophil	-0.18	0.58	-0.53	0.07	-.118	0.71
Eosinophil	0.19	0.55	0.31	0.32	.056	0.86
INR	-0.01	0.95	0.18	0.27	.293	0.07
Prothrombin Time	0.01	0.92	0.16	0.32	.239	0.14
D-dimer	-0.09	0.67	0.36	0.09	.077	0.72
C-Reactive Protein	0.11	0.52	-0.18	0.29	-0.14	0.43
Alanine transaminase (ALT)	-0.34*	0.005*	0.35*	0.005*	-.034	0.82
Aspartate Transaminase (AST)	-0.53*	0.001*	0.57*	0.001*	.014	0.92
Albumin	-0.14	0.34	-2.25	0.08	-.239	0.10
Bilirubin	-0.12	0.58	-0.23	0.29	.189	0.39
Serum. creatinine	-0.29*	0.01*	0.27*	0.02*	-.035	0.81
Lactate dehydrogenase (LDH)	0.14	0.52	-0.06	0.77	.071	0.74
PH	-0.09	0.50	-0.19	0.19	-.091	0.54
PCO2	-0.02	0.90	0.05	0.72	.049	0.74
PO2	-0.08	0.59	0.06	0.69	-.082	0.58
HCO3	0.05	0.72	0.01	0.93	-.058	0.70

RBG, random blood glucose; RBCs, red blood cells; HB, hemoglobin; MCV, mean corpuscular volume. MCH, Mean Corpuscular Hemoglobin; MCHC, Mean Corpuscular Hemoglobin Concentration; RDW, Red Cell Distribution Width; WBCs, white blood cells.

### Sensitivity and specificity of *NEAT-1*, and *IL-6*, *miR374b-5p* level in diagnosing COVID-19 cases

**Fig 5:**
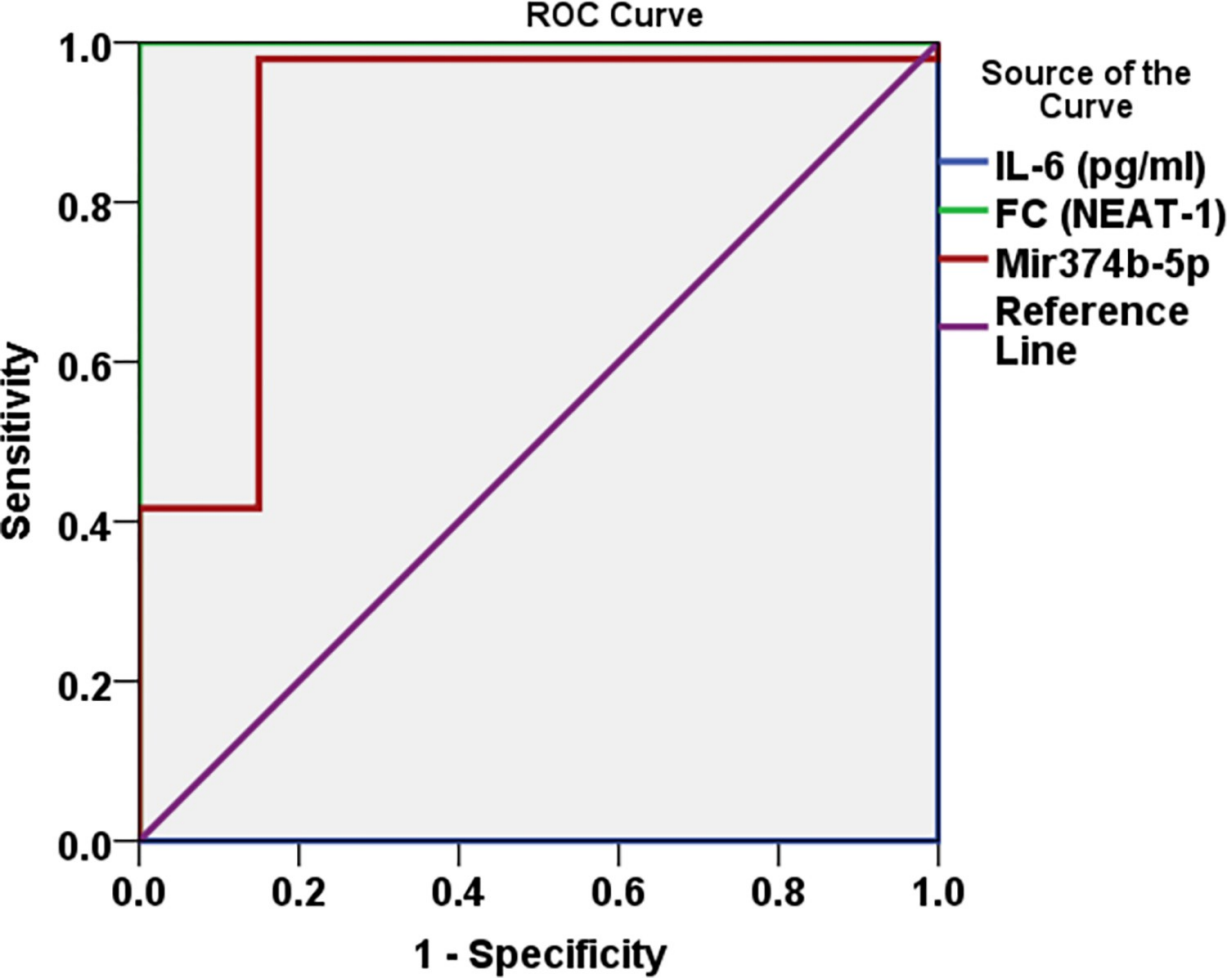
ROC curve for *NEAT-1* and *IL-6*, *miR 374b-5p* levels in the diagnosis of cases.

Sensitivity and specificity tests for *NEAT-1* and *IL6* levels in the diagnosis of cases illustrated a sensitivity of (100% and 97.9%) and a specificity of (85% and 100%) at cut-off values (of 0.985 and 12.55) respectively. At the same time, *miR374b-5p* showed sensitivity and specificity of around 85%in distinguishing COVID-19 patients from controls (**Table 9, Fig 5**).

**Table 9:** Sensitivity and specificity of *NEAT-1*, *IL-6*, and *miR374b-5p* in the diagnosis of cases.

Variable	AUC 95% CI	p-value	Sensitivity	Specificity	Accuracy	Cut off point
**FC (*NEAT-1*)**	1(1-1)	.000	100%	85%	100%	0.985
***IL-6* (pg/ml)**	1(1-1)	.000	97.9%	100%	100%	12.55
**FC *miR374b-5p***	0.895(0.796-0.993)	.000	91.7%	85%	89.5%	0.81

AUC is the area under the curve; CI is the confidence interval. *Significant at P<0.05.

### 7. Relationship between radiological severity score (CXR and CT-TSS) of SARS-CoV2 and three target genes (*NEAT-1*, *miR374b-5p*, and *IL6*)

No significant association was detected between target genes and radiological severity indices (CXR and CT-TSS) **(Table 10A and 10B)**.

**Table 10:** A, B. Relationship between radiological severity score (CXR and CT-TSS) of SARS-CoV2 and three target variables (*NEAT-1*, *miR374b-5p*, and *IL6*).

CXR score		Frequency	Percent	**CT.TSS**		Frequency	Percent
Group	non	2	4.8	Valid	non	1	2.1
	mild	15	35.7		mild	11	22.9
	moderate	19	45.2		moderate	24	50.0
	sever	6	14.3		severe	12	25.0
	Total	42	100.0		Total	48	100.0

CXR score		Mean	Std. Deviation	P value	CT.TSS			Mean	Std. Deviation	P value
FC (*NEAT-1)*	non	.03796	.020489	.831	FC (*NEAT-1*)	non		.08944	.	.885
	mild	.13021	.155298			mild		.12474	.162898	
	moderate	.11927	.132238			moderate		.10197	.073055	
	sever	.11068	.057779			sever		.13369	.162705	
	Total	.11808	.129250			Total		.11485	.121101	
*IL-6* (pg/ml)	non	35.400	4.6669	.511	*IL-6* (pg/ml)		non	43.100	.	.953
	mild	60.727	28.1158				mild	61.964	36.4116	
	moderate	70.337	97.5092				moderate	71.633	85.5441	
	sever	138.767	252.7325				sever	85.283	179.5844	
	Total	75.017	113.9753				Total	72.235	107.2266	
*Mir374b-5p*	non	.0400	.02828	.328	*FC (Mir374b-5p*		non	.0400	.	.888
	mild	.8707	1.76712				mild	.2755	.29981	
	moderate	.2589	.26337				moderate	.5225	1.43277	
	sever	.1517	.12090				sever	.3325	.32241	
	Total	.4517	1.09610				Total	.4083	1.03110	

## Discussion

The SARS-CoV-2 pandemic poses an enormous threat to global public health due to its high morbidity and mortality. Disruption of cytokine regulation caused by COVID-19 is known as a cytokine storm and culminates in multiple organ failure and acute respiratory distress syndrome. Therefore, developing therapeutic strategies with minimal side effects is imperative in the fight against this virus. Preventing patient deterioration and preserving life requires efficient suppression of cytokine storms [7].

Mammalian cells express noncoding RNAs (ncRNAs) extensively, and these molecules serve as important RNA regulators in a range of cellular processes, including the activation of inflammatory signaling pathways. Infections caused by RNA or DNA viruses have been found to regulate the expression of numerous ncRNAs. Hence, viral regulation of ncRNAs, in turn, affects the expression of pro-inflammatory molecules [7].

The SARS-CoV-2 virus’s frequent mutations have made disease control with vaccines and antiviral drugs difficult as newer variants emerge regularly. As a result, there is a need for more effective coronavirus drugs. Hence, detecting the expression of various ncRNA in SARS-CoV2 implies therapeutic strategies for the disease [6].

In our study, we focus on measuring the expression levels of two ncRNAs (*NEAT-1* and *miR-374b-5p*) due to the following causes: both *NEAT-1* and *miR374b-5p* are pro-inflammatory molecules that affect innate immune responses, which is the first line of defense of the host against viral infection [6,9]. Furthermore, *NEAT-1* has been reported to function as a competing endogenous RNA (ceRNA) by competitive binding to *miR-374b-5p* [9]. Finally, ***Morenikeji et al*. *2022*** bioinformatic analysis conveyed that miR374b is a promising antiviral drug that inhibits enzymes crucial for the virus life cycle [22]. Also, we aimed to measure serum *IL6* and explore its correlation to *NEAT-1* and *miR374b-5p*. *IL6* is a crucial player in the cytokine story [14] and a target for both *NEAT-1* and *miR374b-5p*[10].

Our findings revealed for the first time a significant decrease in *NEAT-1*-fold change in the sera from COVID-19 patients. Furthermore, it was the first study to measure serum *miR374b-5p* and found a lower level of *miR374b-5p*, while we found a significant increase in *IL6* in the blood of COVID-19 patients compared to controls.

Regarding *NEAT-1*, these finding agrees with ***Meydan et al*., *2020*** who demonstrated that bioinformatic analysis of differentially expressed ncRNAs in the RNA-seq datasets from lung, brain, and blood of COVID-19 inflammation-prone individuals revealed downregulation of *NEAT-1* and *DANCER* in infected lung cell with COVID19 in contrary to lung cell infected with H1N1 pandemic (influenza virus) which doesn’t affect the expression level of *NEAT-1* and *DANCER* [6]. In contrast to our results, many researchers reported that *NEAT-1* was highly expressed in various samples from COVID-19 patients, such as saliva, serum, whole blood, lung cells [4,8,7,23].

Previous studies reported that *NEAT-1* was among the most differentially expressed lncRNAs in SARS-CoV2 and played a crucial role in cytokine storm formation through different mechanisms by inducing pro-inflammatory cytokines such as *IL6*, *IL8*, and *TNF-α*, which are essential players in the innate immune response to SARS-CoV-2 infection [6]. Also, *NEAT-1* can regulate inflammatory-related genes (*HIF1a*, *CCR7*, and *TLR4*) in SARS-CoV2 patients, and their transcript levels correlate positively with the disease’s severity[6]. *NEAT-1* activates caspase-1 by binding to pro-caspase-1 and activating *NLRP3*, *NLRC4*, and *AIM2* inflammasomes, increasing cytokine production and pyroptosis. Furthermore, *NEAT-1* has been shown to act as "sponges" in mediating inflammation by blocking the activity of "sponged" miRNAs, e.g., *miR342-3p* [24], *miR124*, and *mir129*[25]. *MiR-3076-3p* is absorbed by *NEAT-1* and reduced, which controls the expression of *NLRP3*. Upregulated *NEAT-1* competes with *Let-7a* to release *TLR4*, activating it and stimulating downstream signaling. *NEAT-1* competitively binds *miR-1246* and releases *NAKP*, which mediates *TNF-α* and *IL-1*-induced NF-κB activation and severe inflammatory response [24,25]Click or tap here to enter text.

In COVID-19, *IL-6* is frequently described as a pro-inflammatory cytokine that plays an important role in pathogen resistance and tissue homeostasis. *IL-6* concentrations in normal human serum are typically low (1-5 pg/ml). At the pandemic’s beginning, elevated cytokine levels (notably *IL-6*, *GM-CSF*, *TNF*, interferons, and *IL-18*) were commonly reported in severely ill patients with COVID-19, contributing to the cytokine storm [11].

The down-regulated *miR374b-5p* reported in this study agrees with bioinformatic analysis that reveals that this down-regulation is determined in all stages of the disease [26]. *MiR374b* was one of 27 miRNAs discovered useful as broad-spectrum antiviral drugs against coronaviruses by inhibiting RNA-dependent RNA polymerase (RdRp), an enzyme required for the coronavirus life cycle. Inhibiting the expression of the RdRp enzyme via noncoding RNA is novel and of great therapeutic importance in controlling coronavirus replication. It could serve as a broad-spectrum antiviral drug. [27].

In the current study, the expression level of *NEAT-1* showed a significant negative correlation with the expression of *IL6*, which showed inconsistency with previous studies [10]. The explanation of these findings may refer to the presence of multiple factors, including several ncRNAs that affect the *IL6* level in COVID-19 patients [28].

However, in this study, the correlation between *miR374b-5p* and *NEAT-1* or *IL6* was insignificant, which needs further functional large-scale studies to be proven. Estimated expression levels of target genes and proteins in the blood of patients with COVID-19 in this study (*NEAT-1*, *miR374b-5p*, and *IL6*), together with the previously reported role of *NEAT-1* and *IL6* in the development of cytokine storms, and the recently reported function of *miR374b-5p* in inhibiting viral replication, may set the foundations for new therapeutic regimens.

Our study explored the relationship between studies genes and the radiological severity score of coronavirus patients and revealed non-significant results. However, there was a statistically significant lower median of *NEAT-1* level among cases with GGO (median (range) was 0.06 (0.001-0.11) versus 0.09 (0.01-0.60) in cases with no GGO (P = 0.01), and higher *IL-6* level among cases with grade 4 CO-RADs degree (62.9 versus nearly 40 for grade 3 and 5) with P-value = 0.04 needed further studies.

The strengths of our study include that it is the first time to detect decreased *NEAT-1* in the serum of COVID-19 patients and the first study to measure serum *miR374b-5p* in COVID-19 patients and to correlate its level with *NEAT-1*, *IL6*, and other laboratory findings. Furthermore, the *NEAT-1*, *miR374b*, and *IL6* axis and the relationship between three genes, radiological findings, and radiological severity scores were validated. There are several limitations, like relatively small patient numbers collected from the same geographical area, which elicit the need for further large-scale functional studies to reveal the mechanistic basis of linking these molecules to the pathogenesis of the disease.

## Conclusions

Our study is the first to detect decreased *NEAT-1* and *miR374b-5p* expression in COVID-19 patients’ serums. There was also an increase in *IL6* levels. There is a negative correlation between *NEAT-1* and *IL6* in COVID-19 patients.

## Supporting information

S1 Text The supplementary file details the basic medical history, full examination, laboratory findings, and treatment regimens for COVID-19 patients. (XLSX)

S2 Text The supplementary file showed the Excel sheet of the raw data of COVID-19 patients and controls. (DOCX)

## References

[1] “Coronavirus disease (ILK) Epidemiological Updates and Monthly Operational Updates”.

[2] Statello L., Guo C. J., Chen L. L., and Huarte M., "Gene regulation by long noncoding RNAs and its biological functions," Feb. 01, 2021, Nature Research. doi: 10.1038/s41580-020-00315-9 33353982 PMC7754182

[3] Pan Y. et al., "Novel Insights into the Emerging Role of Neat1 and Its Effects Downstream in the Regulation of Inflammation," 2022, Dove Medical Press Ltd. doi: 10.2147/JIR.S338162 35115805 PMC8802408

[4] Tayel S. I., El-Masry E. A., Abdelaal G. A., Shehab-Eldeen S., Essa A., and Muharram N. M., "Interplay of LncRNAs NEAT1 and TUG1 in Incidence of Cytokine Storm in Appraisal of COVID-19 Infection," Int J Biol Sci, vol. 18, no. 13, pp. 4901–4913, 2022, doi: 10.7150/ijbs.72318 35982898 PMC9379411

[5] Huang K., Wang C., Vagts C., Raguveer V., Finn P. W., and Perkins D. L., "Long noncoding RNAs (lncRNAs) NEAT1 and MALAT1 are differentially expressed in severe COVID-19 patients: An integrated single-cell analysis," PLoS One, vol. 17, no. 1 January, Jan. 2022, doi: 10.1371/journal.pone.0261242 35007307 PMC8746747

[6] Meydan C., Madrer N., and Soreq H., "The Neat Dance of COVID-19: NEAT1, DANCR, and Co-Modulated Cholinergic RNAs Link to Inflammation," Front Immunol, vol. 11, Oct. 2020, doi: 10.3389/fimmu.2020.590870 33163005 PMC7581732

[7] Rahni Z. et al., "Long noncoding RNAs ANRIL, THRIL, and NEAT1 as potential circulating biomarkers of SARS-CoV-2 infection and disease severity," Virus Res, vol. 336, Oct. 2023, doi: 10.1016/j.virusres.2023.199214 37657511 PMC10502354

[8] Abbasi-Kolli M. et al., "The expression patterns of MALAT-1, MIR, THRIL, and miR-155-5p in the acute to the post-acute phase of COVID-19 disease," Brazilian Journal of Infectious Diseases, vol. 26, no. 3, May 2022, doi: 10.1016/j.bjid.2022.102354 35500644 PMC9035361

[9] Huang F., Su Z., Yang J., Zhao X., and Xu Y., "Downregulation of lncRNA NEAT1 interacts with miR-374b-5p/PGAP1 axis to aggravate the development of osteoarthritis," J Orthop Surg Res, vol. 18, no. 1, Dec. 2023, doi: 10.1186/s13018-023-04147-z 37691099 PMC10494329

[10] Xiang M. et al., "LncRNA NEAT1 promotes IL-6 secretion in monocyte-derived dendritic cells via sponging miR-365a-3p in systemic lupus erythematosus," Epigenetics, vol. 18, no. 1, 2023, doi: 10.1080/15592294.2023.2226492 37343193 PMC10286691

[11] Broman N. et al., "IL-6 and other biomarkers as predictors of severity in COVID-19," Ann Med, vol. 53, no. 1, pp. 410–412, 2021, doi: 10.1080/07853890.2020.1840621 33305624 PMC7935117

[12] Chen L. Y. C., Hoiland R. L., Stukas S., Wellington C. L., and Sekhon M. S., "Confronting the controversy: Interleukin-6 and the COVID-19 cytokine storm syndrome," Oct. 01, 2020, European Respiratory Society. doi: 10.1183/13993003.03006-2020 32883678 PMC7474149

[13] Santa Cruz A. et al., "Interleukin-6 Is a Biomarker for the Development of Fatal Severe Acute Respiratory Syndrome Coronavirus 2 Pneumonia," Front Immunol, vol. 12, Feb. 2021, doi: 10.3389/fimmu.2021.613422 33679753 PMC7930905

[14] Jones S. A. and Hunter C. A., "Is IL-6 a key cytokine target for therapy in COVID-19?," Jun. 01, 2021, Nature Research. doi: 10.1038/s41577-021-00553-8 33850327 PMC8043092

[15] Vatansever H. S. and Becer E., "Relationship between IL-6 and COVID-19: To be considered during treatment," Dec. 01, 2020, Future Medicine Ltd. doi: 10.2217/fvl-2020-0168

[16] Perez-Sanchez C. et al., "miR-374a-5p regulates inflammatory genes and monocyte function in patients with inflammatory bowel disease," Journal of Experimental Medicine, vol. 219, no. 5, May 2022, doi: 10.1084/jem.20211366 35363256 PMC8980842

[17] Zhao L. et al., "Diminished miR-374c-5p negatively regulates IL (interleukin)-6 in unexplained recurrent spontaneous abortion," J Mol Med, vol. 100, no. 7, pp. 1043–1056, Jul. 2022, doi: 10.1007/s00109-022-02178-3 35689099

[18] Prokop M. et al., "CO-RADS: A Categorical CT Assessment Scheme for Patients Suspected of Having COVID-19-Definition and Evaluation," Radiology, vol. 296, no. 2, pp. E97–E104, Aug. 2020, doi: 10.1148/radiol.2020201473 32339082 PMC7233402

[19] de Jaegere T. M. H., Krdzalic J., Fasen B. A. C. M., and Kwee R. M., "Radiological Society of North America chest ct classification system for reporting covid-19 pneumonia: Interobserver variability and correlation with reverse-transcription polymerase chain reaction," Radiol Cardiothorac Imaging, vol. 2, no. 3, Jun. 2020, doi: 10.1148/ryct.2020200213 33778589 PMC7294823

[20] Wasilewski P. G., Mruk B., Mazur S., Półtorak-Szymczak G., Sklinda K., and Walecki J., "COVID-19 severity scoring systems in radiological imaging – A review," 2020, Medical Science International. doi: 10.5114/pjr.2020.98009 32817769 PMC7425223

[21] Ali M. A. et al., "Expression profile of serum LncRNAs MALAT-1 and CCAT-1 and their correlation with Mayo severity score in ulcerative colitis patients can diagnose and predict the prognosis of the disease," Noncoding RNA Res, vol. 9, no. 2, pp. 318–329, Jun. 2024, doi: 10.1016/j.ncrna.2024.01.012 38505308 PMC10945117

[22] Morenikeji O. B. et al., "Deciphering inhibitory mechanism of coronavirus replication through host miRNAs-RNA-dependent RNA polymerase interactome," Front Genet, vol. 13, Aug. 2022, doi: 10.3389/fgene.2022.973252 36092931 PMC9459146

[23] Rodrigues A. C. et al., "NEAT1 and MALAT1 are highly expressed in saliva and nasopharyngeal swab samples of COVID-19 patients," Mol Oral Microbiol, vol. 36, no. 6, pp. 291–294, Dec. 2021, doi: 10.1111/omi.12351 34463043 PMC8661855

[24] Lin Y., Sun Q., Zhang B., Zhao W., and Shen C., "The regulation of lncRNAs and miRNAs in SARS-CoV-2 infection," 2023, Frontiers Media SA. doi: 10.3389/fcell.2023.1229393 37576600 PMC10416254

[25] Pan Y. et al., "Novel Insights into the Emerging Role of Neat1 and Its Effects Downstream in the Regulation of Inflammation," 2022, Dove Medical Press Ltd. doi: 10.2147/JIR.S338162 35115805 PMC8802408

[26] Srivastava S. et al., "Evaluation of altered miRNA expression pattern to predict COVID-19 severity," Heliyon, vol. 9, no. 2, Feb. 2023, doi: 10.1016/j.heliyon.2023.e13388 36743852 PMC9889280

[27] Morenikeji O. B. et al., "Deciphering inhibitory mechanism of coronavirus replication through host miRNAs-RNA-dependent RNA polymerase interactome," Front Genet, vol. 13, Aug. 2022, doi: 10.3389/fgene.2022.973252 36092931 PMC9459146

[28] Zhang J. and Chu M., "Targeting of IL-6-Relevant Long Noncoding RNA Profiles in Inflammatory and Tumorous Disease," Inflammation, vol. 42, no. 4, pp. 1139–1146, Aug. 2019, doi: 10.1007/s10753-019-00995-2 30825076

