# Supplementary material for: Peripheral lncRNA NEAT-1, miR374b-5p, and IL6 panel to guide in COVID-19 patients’ diagnosis and prognosis
Source: PLoS One. 2024 Dec 27;19(12):e0313042. doi: 10.1371/journal.pone.0313042 (PMC11676515; doi:10.1371/journal.pone.0313042)
Supplement: S2 Text — (DOCX) [file pone.0313042.s002.docx]

***Results***

**Table (A): Frequency of medical criteria among the COVID-19 group.**

| **Variables**  (n=48) | **Frequency** | |
| --- | --- | --- |
|  | **No.** | **%** |
| **Comorbidities** |  |  |
| DM | 39 | 81.3% |
| HTN | 23 | 47.9% |
| Coronary disease | 3 | 6.3% |
| CKD | 3 | 6.3% |
| Other comorbidities | 39 | 79.2% |
|  |  |  |
| Inotropes | 7 | 14.6% |
| On Oxygen | 48 | 100% |
| ICU admission | 29 | 60.4% |
| Duration of hospital stay (days) | 9.2±5.5 range (2-25) | |
| **Type of ventilation** | **No.** | **%** |
| PEEP NIV | 7 | 14.6% |
| Mask, reservoir, nasal cannula | 21 | 43.8% |
| MV | 17 | 35.4% |
| CPAP | 3 | 6.3% |

MV; Mechanical ventilation, PEEP-NIV; Positive-End Expiratory Pressure -Noninvasive Ventilation, CPAP; Continuous positive airway pressure.

**Table (B): Description of vital signs and blood gases among cases.**

Table 2 describes the mean **±**SD of vital signs, arterial blood gases, oxygen saturation at baseline and on oxygen, and GCS for all cases.

| **Variables** | **Mean ±SD** | **Range** |
| --- | --- | --- |
| **Vital signs** |  |  |
| SBP | 136.6±27.9 | 60-198 |
| DBP | 81.7±14.7 | 50-115 |
| Temperature | 37.2±0.70 | 36-39 |
| RR | 29±8 | 15-56 |
| HR | 92±23 | 50-150 |
| **ABG** |  |  |
| PH | 7.3±0.15 | 6.8-7.6 |
| PCO2 | 40.3±12.8 | 20.6-78.2 |
| PO2 | 56.2±24.2 | 22.7-136 |
| HCO3 | 21.04±5.9 | 7-31.3 |
| O2 on RA | 84.8±8.6 | 60-97 |
| O2 on oxygen | 93.9±4.7 | 80-100 |
| GCS | 14±3 | 5-15 |

GCS, Glasgow Coma Scale; O2 on RA, oxygen on room air.

**Table (C): Description of laboratory investigations among cases.**

Regarding laboratory investigations of cases, **Table 3** showed normal sodium and potassium levels with high levels of RBG, CRP, ALT, AST, creatinine, and LDH levels, with lower levels of albumin. CBC shows mild anemia. 79.2% of cases show positive PCR tests.

Table 4 illustrated a statistically significant higher mean of WBCS, ALT, AST, and serum creatinine levels with a p-value <0.05 among cases compared to control. On the other hand, there was no statistically significant difference with p-value >0.05 between cases and controls as regards hemoglobin level and PLT count.

| **Variables** | **Cases (N=48)** | **Control (N=40)** | **P-value** |
| --- | --- | --- | --- |
|  | **Mean ±SD** | **Mean ±SD** |  |
| Serum Na | 139.8±9.1 |  |  |
| Serum K | 7.03±1.2 |  |  |
| RBG | 317.9±160.9 |  |  |
| RBCS | 4.4±0.73 |  |  |
| HB (g/dl) | 11.9±2.3 | 13.1±1.3 | 0.31 |
| Hematocrit (%) | 36.6±6.6 |  |  |
| MCV (fl) | 83.6±6.9 |  |  |
| MCH (pg) | 27.2±2.9 |  |  |
| MCHC (g/dl) | 32.6±1.4 |  |  |
| RDW (%) | 14.2±2.03 |  |  |
| Platelet Count (10^9^/L). | 215.4±80.8 | 213.2±51.4 | 0.53 |
| Mean plat. Volume | 9.2±1.1 |  |  |
| Total WBCs (10^9^/L). | 9.2±5.9 | 5.7±1.5 | **<0.001*** |
| Neutrophil | 7.5±6 |  |  |
| Lymphocyte | 0.94±0.46 |  |  |
| Monocyte | 0.39±0.33 |  |  |
| Basophil | 0.01±0.004 |  |  |
| Eosinophil | 0.01±0.01 |  |  |
| INR | 1.4±0.50 |  |  |
| PT | 19.7±9.3 |  |  |
| D-dimer (ng/mL) | 0.46±0.50 |  |  |
| CRP (mg/L) | 49.7±30.8 |  |  |
| ALT (U/L) | 83.8±124.7 | 20.7±6.7 | **<0.001*** |
| AST (U/L) | 87.6±120.9 | 15.7±7.2 | **<0.001*** |
| Albumin (g/dl) | 2.9±0.49 |  |  |
| Bilirubin (mg/dl) | 0.67±0.34 |  |  |
| S. creatinine (mg/dl) | 1.8±1.9 | 0.93±0.21 | **0.004*** |
| LDH | 494.4±295.7 |  |  |
| **1^st^ PCR** | **NO** |  |  |
| Negative | 10 |  |  |
| Positive | 38 |  |  |

**Table (D): Frequency of CT radiological findings among the patient group.**

Table 4 illustrates that 93.8% of cases show bilateral GGO, with 58.3% having mixed GGO and consolidation and 41.7% show nodules. For lesion distribution in CT, 52.1% of cases show mixed distribution. 64.6% of cases show grade 5 in CT **CO-RADS** degree, 83.3% had typical CT chest findings (CT- RSNA), The percentage of lung affected with GGA was (47.6±16.5), with mean affected lobes of (5±0.55), and mean TSS total score was (8.8±3.9), and finally mean CXR score 18 was (8.1±4.03)

| **Variables**  (n=48) | **Frequency** | |
| --- | --- | --- |
|  | **Number** | **%** |
| **CT findings** |  |  |
| Bilateral GGO | 45 | 93.8% |
| Rt lung affection | 3 | 6.3% |
|  |  |  |
| GGO | 15 | 31.3% |
| Consolidation | 4 | 8.3% |
| Mixed GGO and consolidation | 28 | 58.3% |
| Plural effusion | 16 | 33.3% |
| Nodules | 20 | 41.7% |
| **Distribution** |  |  |
| Central distribution | 7 | 14.6% |
| Peripheral distribution | 13 | 27.1% |
| Mixed distribution | 25 | 52.1% |
| **CO-RADS degree** | **No.** | **%** |
| Grade 2 | 1 | 2.1% |
| Grade 3 | 4 | 8.3% |
| Grade 4 | 12 | 25% |
| Grade 5 | 31 | 64.6% |
| **CT-RSNA** |  |  |
| Atypical | 1 | 2.1% |
| Typical | 40 | 83.3% |
| Undetermined | 7 | 14.6% |
|  | **Mean ±SD** | **Range** |
| Percentage of GGA | 47.6±16.5 | 8-80 |
| Number of affected lobe | 5±0.55 | 2-5 |
| TSS total score | 8.8±3.9 | 2-17 |
| CXR score 18 | 8.1±4.03 | 0-15 |

GGO; ground-glass opacity, TSS, total severity score (range from 0 to 20), RSNA, Radiological Society of North America Chest CT Classification System; CXR score 18, Modified Chest X-Ray Scoring System in Evaluating Severity of COVID-19 (range from 0 to 18); CO-RADS COVID-19 Reporting and Data System.

**Table (E): Frequency of types of treatment used among the patient group.**

Table 5 shows treatment regimens; 29.2% were treated with Remedesevir versus 10.4% receiving Ivermectin, and only 2.1% were treated with either Favipiravir or Ribavirin.

| **Variables**  (n=48) | **Frequency** | |
| --- | --- | --- |
|  | **No** | **%** |
| Ivermectin | 5 | 10.4% |
| Remedesevir | 14 | 29.2% |
| Favipiravir | 1 | 2.1% |
| Ribavirin | 1 | 2.1% |
